# The ankle brachial index in people with and without diabetes: intra-tester reliability

**DOI:** 10.1186/s13047-020-00389-w

**Published:** 2020-05-12

**Authors:** Sarah Louise Casey, Sean Michael Lanting, Vivienne Helaine Chuter

**Affiliations:** 1 School of Health Sciences, University of Newcastle, Ourimbah, Australia; 2 Priority Research Centre for Physical Activity and Nutrition, University of Newcastle, Callaghan, Australia

**Keywords:** Ankle-brachial index, Reliability, Peripheral arterial disease

## Abstract

**Background:**

The ankle-brachial index (ABI) is widely used for determining the presence and severity of peripheral arterial disease (PAD), and current guidelines suggest it should be used to monitor possible progression in affected individuals. It is therefore important that the technique demonstrates adequate reliability for repeated measurements. Existing studies suggest that the ABI is reliable in the general population however, there is a lack of evidence for the reliability of the ABI in people with diabetes. The aim of this study was to investigate the intra-tester reliability of the ABI in people with and without diabetes.

**Methods:**

Eighty-five participants (40 with and 45 without diabetes) underwent ankle and brachial systolic blood pressure measurements by a single clinician during two testing sessions. Intraclass correlation coefficients (ICC), their 95% limits of agreement, standard error of measurement and minimal detectable change were determined.

**Results:**

Intra-tester reliability of the ABI was found to be good (ICC: 0.80), however sub-group analysis of participants with and without diabetes found that ABI was slightly less reliable in people with diabetes (ICC: 0.78) than in those without (ICC: 0.82). The relatively large limits of agreement (− 0.16 to 0.16), standard error of measurement (0.03 overall, 0.04 for the diabetes group), and minimal detectable change (0.08 overall, 0.11 for the diabetes group) suggest that a large change in ABI is required for it to demonstrate a true change rather than the result of measurement variability. The minimal detectable change for the ABI was 0.08 overall, and 0.11 for the diabetes group.

**Conclusions:**

The ABI demonstrated good reliability in all groups analysed. However, the wide limits of agreement and considerable standard error of measurement obtained support the use of multiple methods of vascular assessment for ongoing monitoring of lower limb vascular status.

## Background

Peripheral arterial disease (PAD) is estimated to affect up to 12% of the general population [1], and up to 14% of people with diabetes [2]. Characterised by progressive stenosis of the lower limb arteries, the presence of PAD is strongly predictive of morbidity and mortality in people with diabetes [3].

Due to the high risk of concurrent cardiovascular disease, and lower limb complications associated with PAD, and the rapid progression of atherosclerosis in people with diabetes, accurate and reliable diagnostic testing methods are necessary for early diagnosis and effective ongoing monitoring. Early detection of PAD allows for intervention and management of coexisting cardiovascular disease, including lifestyle advice and medication [4, 5], and prevention and management of lower limb complications such as foot ulceration and amputation [6].

The ankle brachial index (ABI) is typically calculated as the ratio of the highest of the dorsalis pedis and posterior tibial artery systolic pressure to the highest of the left and right brachial systolic pressures [7]. A normal value for ABI assessment within the general population is considered to be 1.00–1.40 [8], with values 0.91–0.99 classed as ‘borderline’, while those below 0.91 represent likely PAD [9]. The ABI is a widely used as a non-invasive method of conducting objective lower limb vascular assessment to monitor at risk patients for the development of PAD [9]. Current international guidelines recommend the ABI be used routinely to monitor for PAD in older people, those with a smoking history and in people with diabetes [8, 9].

Due to the requirement for periodic assessments over the long term to identify development, and where relevant, progression of PAD, high reliability of the ABI is essential [7, 10]. There are a number of factors that are recognised to affect ABI measurement, and, subsequently, its reliability. These include recent caffeine ingestion [11], tobacco use [12] or exercise [13, 14], clinical experience [15, 16], as well as measurement procedure including lack of pre-test rest [17] and body position [18] during measurement. Existing studies of ABI reliability are inconclusive, with inconsistencies in methodology, statistical analysis and reporting making interpretation of the findings difficult, particularly when coupled with the lack of data for those without symptoms, but for whom screening is recommended [19]. There are currently little available data quantifying the magnitude of error that occurs with the measurement and therefore the amount of change in ABI that represents a true change (over and above measurement error) [20]. There has also been limited investigation of the intra-tester reliability of the ABI in people with diabetes, with only one previous study, which reported a coefficient of variation of 8% [21]. In diabetes cohorts, PAD is known to have a more aggressive disease presentation [22], to be more likely to affect infra-genicular arteries [23] and to co-exist with medial arterial calcification which can prevent compression and pressure measurement of the lower leg arteries [24]. Although there is significant evidence that these factors affect diagnostic accuracy of the ABI in this population, it is unknown the extent to which the presence of diabetes may affect ABI reliability [25–27]. The primary aim of this study was to determine the intra-tester reliability of the ABI in adults with and without a diagnosis of type 1 or type 2 diabetes mellitus.

## Methods

This reliability study was undertaken at the University of Newcastle Podiatry Clinic on the New South Wales Central Coast. Ethics approval was obtained from the University of Newcastle Human Research Ethics Committee (H-2010-1230).

### Participants

Participants were recruited on a volunteer basis from the current patient population attending the University of Newcastle Podiatry Clinic and the local community, via flyer advertising. Informed written consent was given by all participants prior to their participation in this study. Inclusion criteria were in line with the current American Heart Association (AHA) recommendations for PAD screening using resting ABI [9]: 65 years of age and over, or aged 50 years and over with risk factors for atherosclerosis (e.g. diabetes mellitus, history of smoking), or in individuals with exertional leg pain or non-healing wounds. Exclusion criteria were inability to give informed consent, contraindications to brachial and/or ankle pressure measurement including history of deep vein thrombosis, lymphoedema of the arms or legs, history of mastectomy or current leg ulceration preventing placement of a blood pressure cuff around the lower leg, and inability lie in a supine position for 40 min. Participant medical history was obtained through medical records and via self-report.

### Equipment

Systolic ankle pressures were measured using an 8 Hz Bidop ES-100 V3 hand-held Doppler (Hadeco), an adult standard, adult large or adult extra-large inflatable cuff (Liberty Health Care) and an ERKA sphygmomanometer (Kallmeyer Medizintechnik GmbH and Co). Cuff size was determined in accordance with AHA recommendations for cuff width and length [7]. All blood pressure gauges were newly calibrated.

### Procedure

Data collection was performed at the University of Newcastle Podiatry Clinic. All testing was performed by the same clinician, a Podiatrist with more than 15 years’ clinical experience, using ABI several times a week. Participants were asked to avoid alcohol, exercise and caffeine for 1 h prior to participating in each testing session. Participants attended two testing sessions, with the second session occurring 7–10 days after the first, and at a similar time of day (morning or afternoon). The test procedure was carried out in accordance with the American Heart Association Scientific Statement [7]. Tests were performed in a controlled environment with room temperature maintained at 24–25 °C via air-conditioning setting. The participant was maintained in a supine horizontal position for 10 min prior to, and during testing, and was encouraged to rest and not engage in conversation or other activity during the testing process. Blood pressures were measured at the brachial, posterior tibial and dorsalis pedis arteries in both left and right limbs. All blood pressures were measured once, with a minimum of 3 min between pressure measurements. The order of pressure measurements was determined using a computer-generated random allocation. One limb for each participant was selected at random for inclusion in statistical analysis. The ABI was calculated as the ratio of the higher of the dorsalis pedis and posterior tibial systolic blood pressures to the higher of the left or right brachial systolic blood pressures.

### Statistical analysis

Statistical analysis was performed using the Statistical Package Social Science software version 25 (SPSS Inc., Chicago, Illinois). A sample size of a minimum of 40 participants for each group (diabetes and no diabetes) was determined, based on α = 0.5, β = 0.2 for two observations per participant, with a target ICC of 0.8 and a lowest acceptable ICC of 0.6 [28]. It was conservatively anticipated there would be a 15% loss to follow up requiring enrolment of 90 participants. Intra-class correlation coefficient (ICC) estimates and their 95% confidence intervals (CI) were calculated (Model 3,1) based on a two-way mixed effects model to determine level of agreement between test and re-test for the measured ABI. All ICC values were interpreted according to cut-offs suggested by Portney and Watkins [29],in which > 0.75 denotes good reliability, 0.50 to 0.75 suggests moderate reliability, and values below 0.50 represents poor reliability. Paired t-tests were performed for the ABI test and re-test reliability to determine whether a statistically significant difference existed between scores. Measurement error (i.e. the spread of values obtained around the true value) was expressed in the original units using the standard error of measurement, SEM = SD*√(1-ICC) and 95% limits of agreement (LOA) were calculated, 95% LOA = Mean ± SD (1.96) [29]. To determine the smallest amount of change that must be achieved to reflect a true change, outside the error of the tests, the minimal detectable change (MDC) was calculated as MDC = 1.96*SEM*√2 [29]. Independent t-tests were performed on mean brachial and ankle pressures and the mean ABI between the group with diabetes and the group without diabetes to determine the existence of statistically significant differences between the two groups. A Chi-square test was used to test for between group differences for categorical variables including smoking history and gender. For the purposes of this study, ABI values of ≤0.90 and > 1.40 were considered abnormal, with those between 0.91 and 1.40 deemed to be normal.

## Results

Ninety participants were recruited (44 male, 46 female), 45 with diabetes. Of those recruited 5 participants (4 males, 1 female, all with diabetes) were unable to attend follow-up testing on the correct day or time and were excluded from the analysis. Eighty-five participants attended both sessions (40 male, 45 female), 40 with diabetes. The mean age of participants with diabetes was 68 years (SD ±7.3 years), and 76.3 years (±7.4 years) for those without. Mean BMI for those with and without diabetes was 33.9 ± 5.4 and 29.4 ± 3.9 respectively. Participant characteristics are described in Table 1. There was no significant difference between the number of participants with a history of smoking, or between the gender distribution in those with and without diabetes.

**Table 1:** Participant characteristics

	Diabetes (***n*** = 40)	No diabetes (***n*** = 45)	Between group differences
Males n (%)	20 (50)	20 (44)	0.79
Females n (%)	20 (50)	25 (55)	
Age range (years)	50–81	65–94	
Mean age (years)	68.0	76.3	0.001
Mean body mass index	33.9	29.4	0.001
Smoking history n (%)	16 (40)	15 (33)	0.94
Mean diabetes duration (years)	8.1		
Oral hypoglycaemics n (%)	31 (78)		
Insulin n (%)	14 (35)		
Neuropathy n (%)	14 (35)		
Mean ankle pressure 1 mmHg (SD)	129.9 (15.5)	132.7 (11.8)	0.22
Mean ankle pressure 2 mmHg (SD)	132.5 (16.1)	127 (12.6)	0.65
Mean brachial pressure 1 mmHg (SD)	123.6 (11.4)	130.9	0.22
Mean brachial pressure 2 mmHg (SD)	124.7 (11)	129.5	0.10
Mean ABI test 1 mmHg (SD)	1.06 (0.13)	1.04	0.34
Mean ABI test 2 mmHg (SD	1.06 (0.11)	1.04	0.67
Mean ABI ≤0.90 in both assessments	*N* = 3	*N* = 5	

*ABI* ankle brachial index

There was no statistically significant difference between mean ankle pressure, mean brachial pressure or mean ABI values for the two groups (Table 1). The rates of abnormal ABI results across both testing sessions for both groups were low (diabetes *n* = 3, no diabetes *n* = 5). There were no ABI results in excess of 1.4 indicating clinically apparent medial arterial calcification in either group. The diabetes group were significantly younger and with a higher BMI than the group without diabetes. All ICCs were above the lowest acceptable ICC value as previously determined.

Intra-tester reliability results are presented in Table 2. The intra-tester reliability of the ABI was found to be moderate to good, with an ICC value of 0.80 (95% CI 0.70 to 0.86). However, sub-group analysis of participants with and without diabetes found that ABI reliability was slightly lower in participants with diabetes (ICC 0.78, 95% CI 0.62 to 0.88) compared to those without (ICC 0.82, 95% CI 0.70 to 0.90).

**Table 2:** Intra-tester reliability

Measure	ICC	95% CI	Paired t-test ***p*** value	SEM	MDC	95%LOA
*Whole group (N = 85)*						
Ankle pressure	0.89	0.83 to 0.92	< 0.001	2.55	7.07	− 8.69 to 18.22
Brachial Pressure	0.77	0.67 to 0.84	< 0.001	3.31	9.17	−12.27 to 19.12
ABI	0.80	0.70 to 0.86	0.29	0.03	0.08	−0.14 to 0.16
*Diabetes (n = 40)*						
Ankle pressure	0.92	0.85 to 0.96	0.20	1.84	5.10	−15.07 to 10.12
Brachial Pressure	0.84	0.72 to 0.91	0.26	2.51	6.96	−13.37 to 11.12
ABI	0.78	0.62 to 0.88	0.53	0.04	0.11	−0.16 to 0.14
*No diabetes (n = 45)*						
Ankle pressure	0.88	0.80 to 0.93	< 0.001	2.19	6.07	−6.75 to 18.35
Brachial Pressure	0.74	0.58 to 0.85	< 0.001	4.41	12.22	−11.31 to 11.12
ABI	0.82	0.70 to 0.90	0.90	0.03	0.08	−0.15 to 0.15

*ICC* intraclass correlation coefficient, *SEM* standard error of measurement, *MDC* minimal detectable change, *LOA* limits of agreement

## Discussion

The intraclass correlation coefficient (ICC) values obtained suggest that the intra-tester reliability of the ABI is good, with values that are comparable to the findings of previous studies in mixed populations including those with risk factors for and/or suspected PAD [30–32]. Similar values were obtained for the whole group (ICC 0.81) as well as for the group with (ICC 0.78) and without (ICC 0.82) diabetes for all analyses. The limits of agreement (LOA) that were obtained were quite large with upper and lower bounds ranging from − 0.16 to 0.16. The LOA value represents the values between which 95% of the differences between measurements fall, with differences outside of those values being more likely to represent an actual change (rather than measurement error). For this measurement, the LOA suggest that an ABI value of 1.0 could vary from 0.84 to 1.16, and not reflect an actual change in peripheral arterial supply.

Similarly, the standard error of measurement (SEM), is an estimate of how far the sample mean is likely to be from the population mean. A SEM of 0.03 demonstrates that the variability of the test means that a large change in ABI is required clinically for there to be certainty around it being a true change rather than variability of the measurement. This was supported by a large minimal detectable change (MDC), which reflects the amount of change required to indicate a valid change in the test result that is not due to chance [33]. The MDC was found to be 0.08 for the ABI overall, and in participants without diabetes. For the diabetes group, the MDC was 0.11. In practice, this represents a large difference between measures being required to be indicative of clinical change rather than measurement. For example, a decrease of 0.10 in ABI between measurements may represent a change from a normal value of 1.00 to 0.90, indicating a pathological value, however where the MDC is 0.11 this change may occur as a result of measurement variability rather than disease. This finding highlights the need for the results of this test to be interpreted with caution, particularly in people with diabetes. A number of cross sectional studies have identified problems with diagnostic accuracy of the ABI in specific populations, particularly those with diabetes and in older age, and have identified the need for additional testing (e.g. continuous wave Doppler, toe pressures) to be used to provide a more comprehensive evaluation of lower limb status [27, 34]. Although reliability relates to the consistency of the measurement as opposed to the accuracy, our results substantiate the need for multifaceted testing to be used as the variability of the measurement of the ABI likely further affects its effectiveness for identifying and monitoring PAD.

Our sub-analysis by diabetes status demonstrated a small reduction in ABI reliability in those with diabetes. To the authors’ knowledge, there are no published studies that evaluate ABI reliability in people with diabetes using ICC, with which our findings could be compared. However, published studies evaluating the intra-tester reliability of toe brachial index report similar results in people with diabetes (ICC0.72–0.75) compared to those without (ICC 0.74–0.8) [35]. The only previously published study evaluating ABI reliability in people with diabetes reported a coefficient of variation of 8% [21], which is similar to the standard error measurement of ±0.04 found in this study. Diabetes has been associated with several complications that affect diagnostic accuracy of the ABI for PAD including medial arterial calcification (MAC). It is possible such complications could affect reliability, however to our knowledge this has not yet been established. The diabetes cohort recruited to our study, although having increased risk of PAD (e.g. higher BMI, diabetes, insulin use), did not demonstrate overt clinical signs or symptoms of PAD and MAC. This cohort also had a relatively short mean duration of diabetes (< 10 years), making them less likely to have developed related complications. Similarly, the no diabetes group were significantly older (mean age > 75 years) placing them at greater risk of age-related PAD and MAC but lack of abnormally low or high ABI results suggest this was not the case. This may have resulted in the similar reliability of the test in both groups.

Overall brachial pressure measurements were found to be less reliable than ankle pressure measurements in participants with and without diabetes, which is consistent with previous research using handheld Doppler in podiatry practice [35, 36]. The use of automated oscillometric systolic blood pressure measurement has been shown to be more reliable than manual Doppler method in people with diabetes [37]. Given that ankle pressures were found to be highly reliable (ICC 0.88–0.92) improving the reliability of brachial pressure measurement is likely to improve reliability of the ABI overall. Furthermore, the use of automated pressure measurement may reduce the time taken to carry out ABI, which is one of the most frequently cited reasons not to perform vascular assessment [38]. There was a statistically significant difference in ankle pressure and brachial pressure values obtained between the first and second visit, however this was not reflected in a significant difference in the ABI result obtained. This finding may result from variation in the exact timing of the second appointment compared to the first (e.g. early in the morning for visit 1, mid-morning for visit 2), or it may be due to familiarity with the procedure, location and researchers at the second visit.

The findings of this study should be considered in the light of the controlled conditions under which the ABI was performed, which may not reflect those under which the test is performed in clinical practice. Furthermore, all measurements were obtained manually using Doppler, and represent reliability in relation to that technique only. A further limitation is the lack of blinding of the clinician to the pressure values obtained. This study only established the intra-tester reliability of the ABI when assessed by an experienced podiatrist. In clinical practice, the ABI is used by a range of health professionals, and as such the findings of this study may not be generalisable to other clinicians. In addition, the inter-rater reliability of the ABI is of considerable importance, considering that it is often used by different personnel rather than always being repeated by the same clinician. It is also most commonly repeated at intervals of more than 1 week, and not necessarily at the same time of day, and with patients not necessarily having avoided factors that may affect their result.

## Conclusions

The results of this study suggest that while intra class correlation coefficients for the reliability of the ABI are good for people with and without diabetes, measures of the variability of the measurement suggest a large difference between tests results is required to reflect a true change. Therefore, results of the ABI should be considered in the context of other assessment of lower limb vascular status rather than in isolation. As such, our findings support the use of ABI only as part of a comprehensive vascular assessment, with the use of other techniques to form a more complete clinical picture. Further research should evaluate the inter-tester reliability of the ABI and using automated pressure measurement techniques.

## Data Availability

De-identified data is held securely with the senior author.
